# A Case Report on Primary Pulmonary Choriocarcinoma

**DOI:** 10.7759/cureus.63466

**Published:** 2024-06-29

**Authors:** Himanshi Kaushik, Mahesh Deshmukh, Smita Gupte, Niraj Kanchankar, Amol Dongre

**Affiliations:** 1 Oncology, Jawaharlal Nehru Medical College, Datta Meghe Institute of Higher Education and Research, Wardha, IND; 2 Histopathology, Alexis Multispeciality Hospital, Nagpur, IND; 3 Oncology, Datta Meghe Medical College, Datta Meghe Institute of Higher Education and Research, Nagpur, IND; 4 Radiology, Alexis Multispeciality Hospital, Nagpur, IND

**Keywords:** adenocarcinoma, primary pulmonary choriocarcinoma, xx chromosome, lung, choriocarcinoma

## Abstract

Primary choriocarcinoma originating in the lung is a rare entity. These are highly malignant intrapulmonary tumors with a notoriously poor prognosis. The pathogenesis is unclear. A 34-year-old lady, with a history of abortion six months back, presented with left-sided chest pain for one month, dyspnea on exertion, weight loss, and loss of appetite. Computed tomography (CT) of the thorax was suggestive of a mass lesion 4 x 5 cm at the left upper lobe, which was invading the chest wall, and pleural effusion, histopathologically defined as adenocarcinoma. A positron emission tomography-computed tomography (PET-CT) scan showed a fluorodeoxyglucose (FDG) avid lesion in the left upper lobe of size 4 x 5 with invasion to the chest wall with no evidence of distant metastases. Urine pregnancy test (UPT) was negative for this patient. Thus, the patient was initially diagnosed with stage cT3N0M0 adenocarcinoma of left lung cancer. The sample was sent for the lung next-generation sequencing (NGS) panel. Meanwhile, the patient was empirically started on gefitinib. Tumor markers revealed raised beta-human chorionic gonadotropin (β-hCG) to 1,79,000 IU/ml. A review biopsy was done, which was suggestive of choriocarcinoma. Genetic testing of lung biopsy suggestive of XX chromosome, confirming the diagnosis of primary pulmonary choriocarcinoma (PPC). The patient was planned for chemotherapy with etoposide and cisplatin. The patient underwent embolization of the left internal mammary artery (IMA) and branches of the left subclavian vein. There was a gradual fall in β-hCG after the second dose of chemotherapy on day 7. For the diagnosis of PPC, immunohistochemistry (IHC) staining, β-hCG measurement, and examination to exclude primary gonadal malignancies are essential. A combination of surgery and chemotherapy is a favorable treatment. As it's a highly vascular tumor, selective arterial embolization can be life-saving in case of bleeding.

## Introduction

Choriocarcinoma is a germ cell tumor that usually arises in the gonads, particularly the testes or ovaries. Primary choriocarcinoma originating in the lung is an extremely uncommon entity, with only a few cases reported in the literature [1]. Nonspeciﬁc respiratory symptoms, including cough, dyspnea, and chest pain, often mark the clinical presentation of patients harboring these intrapulmonary tumors. Radiological imaging reveals inﬁltrative masses within the lung parenchyma, frequently accompanied by pleural effusion. In many cases, the rapid progression of the disease hinders early detection and intervention, contributing to the dismal prognosis. The scarcity of data on primary pulmonary choriocarcinoma (PPC) has resulted in the absence of an ofﬁcial treatment guideline for this condition. However, existing research indicates that combining surgery and chemotherapy is a promising and favorable approach for managing PPC [2]. This report aims to document the clinical presentation, diagnostic challenges, and aggressive metastatic nature of this rare pulmonary tumor.

## Case presentation

**Figure 1:**
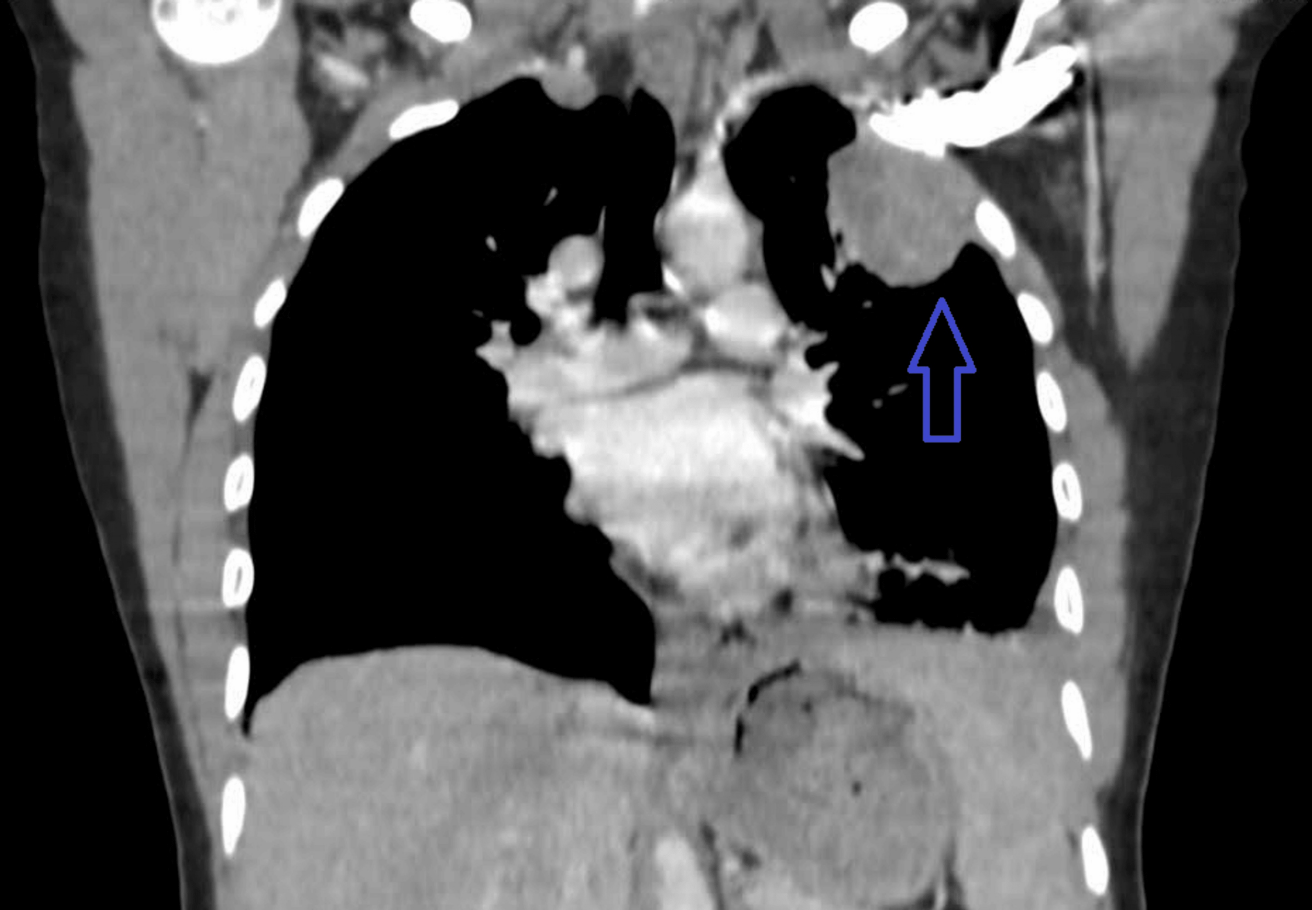
CT scan showing a mass in the left upper lobe CT = Computed tomography

The 34-year-old lady's complex medical presentation began with a constellation of symptoms, including left-sided chest pain persisting for a month, dyspnea on exertion, noticeable weight loss, and a loss of appetite. She also had a missed abortion six weeks prior to the symptoms. Thoracic computed tomography (CT) scan (Figure 1) revealed a substantial 4 x 5 cm mass lesion in the left upper lobe, which invaded the chest wall. The histopathological analysis of the lesion pointed toward adenocarcinoma.

**Figure 2:**
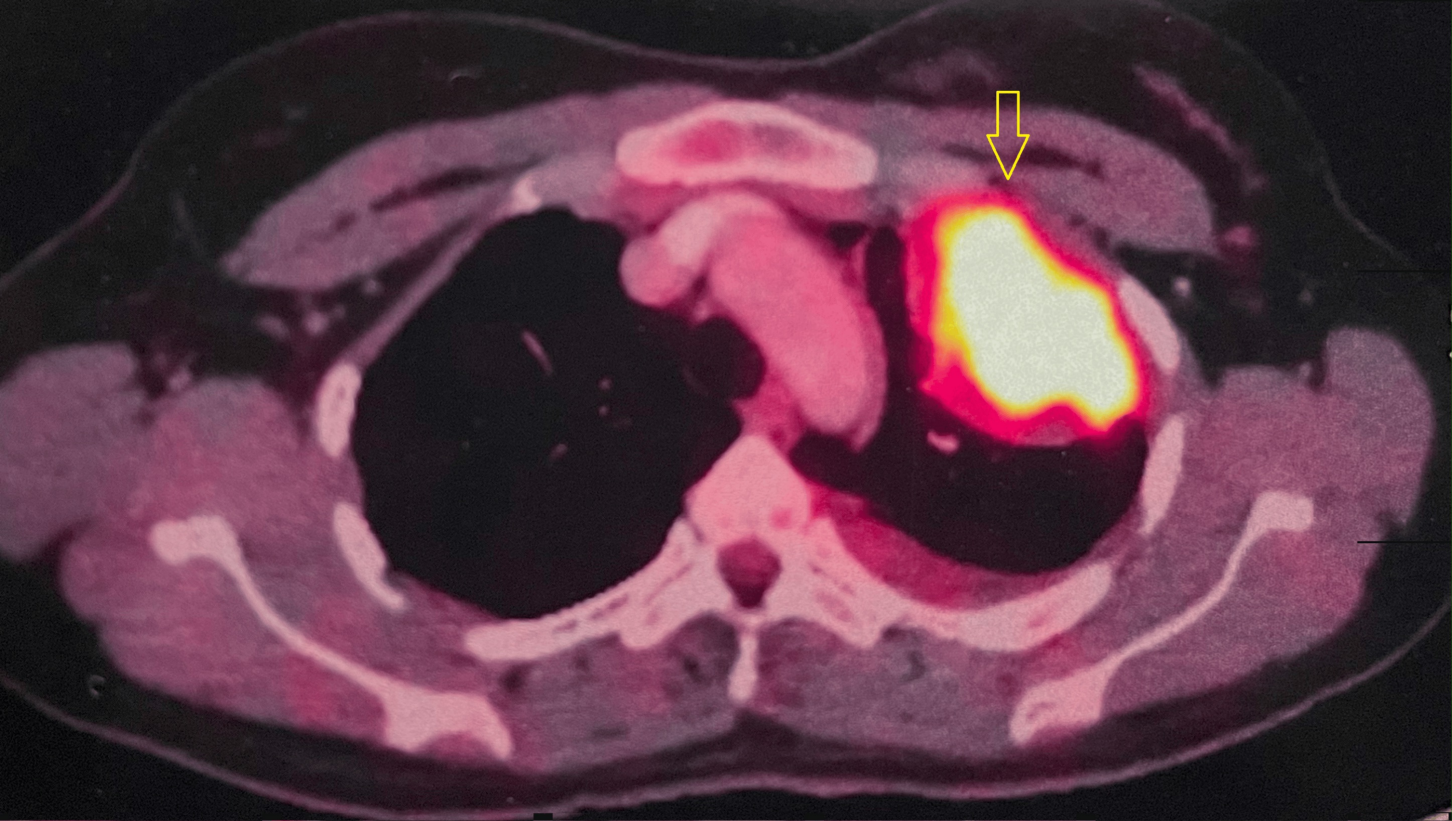
PET-CT scan showing FDG uptake in the left upper lobe PET-CT = positron emission tomography-computed tomography scan; FDG = fluorodeoxyglucose

A comprehensive imaging assessment, including a positron emission tomography-computed tomography scan (PET-CT) scan was conducted to gauge the extent of the disease. The PET-CT results indicated an F-18 fluorodeoxyglucose (FDG) avid lesion in the left upper lobe, with invasive characteristics into the chest wall but, notably, no evidence of distant metastases (Figure 2).

**Figure 3:**
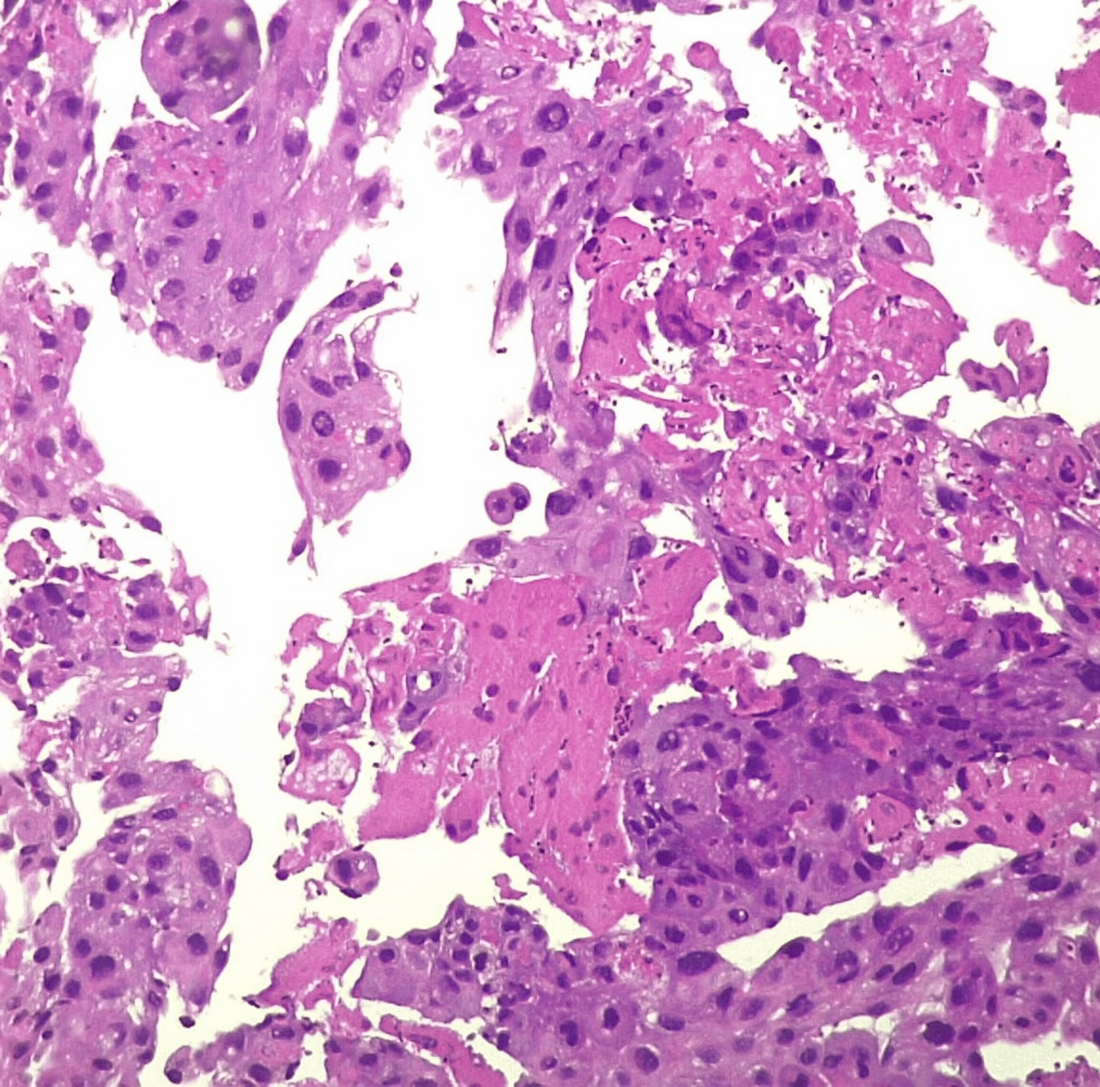
Lung mass biopsy showing sheets of cytotrophoblasts suggestive of choriocarcinoma

**Figure 4:**
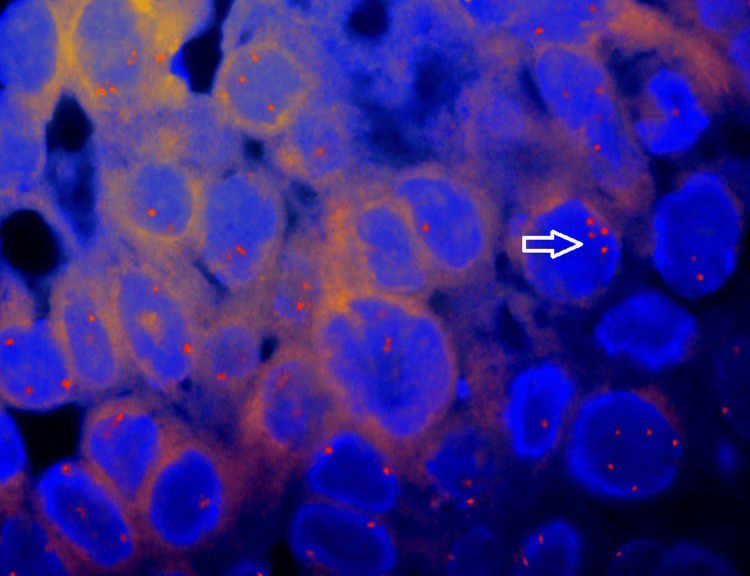
FISH showing 100% female genotype with no visible Y chromosome sequences FISH = Fluorescence in situ hybridization

The initial diagnosis, based on the gathered information, was a stage cT3N0M0 adenocarcinoma of the left lung. In an attempt to address the malignancy, an empirical initiation of geﬁtinib, a tyrosine kinase inhibitor, was pursued. However, an unexpected turn occurred when tumor marker assessments revealed a signiﬁcantly elevated beta-human chorionic gonadotropin (β-hCG) level of 1,79,000 IU/ml (normal range for non-pregnant women < 5 IU/ml), prompting a reevaluation of the diagnosis. A review biopsy, aimed at clarifying the nature of the lesion, pointed toward choriocarcinoma (Figure 3). Adding to the complexity, genetic testing done by fluorescence in situ hybridization (FISH) method conﬁrmed the presence of XX chromosomes, leading to the definitive diagnosis of PPC (Figure 4). 

**Figure 5:**
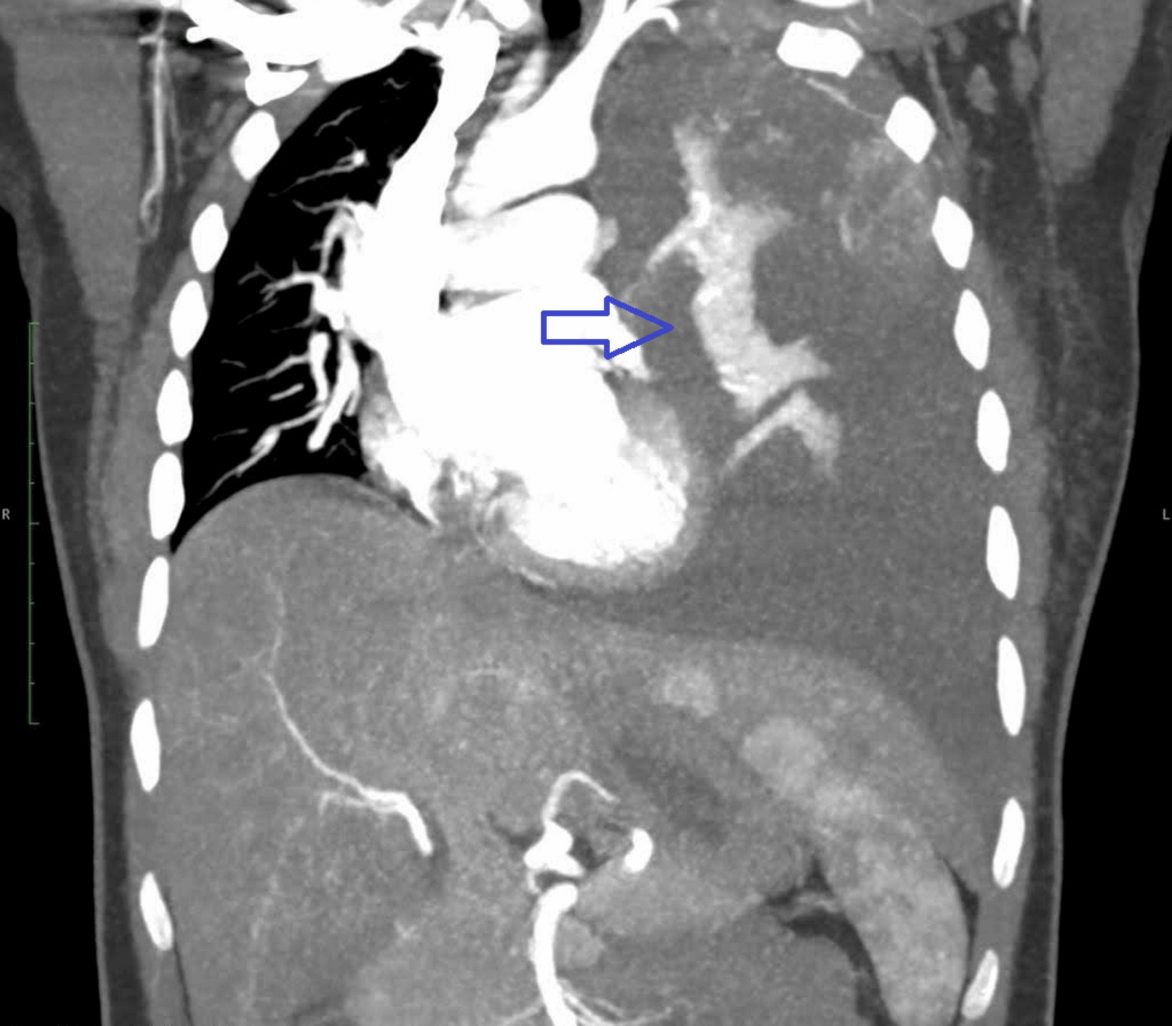
CT angiography CT = Computed tomography

**Figure 6:**
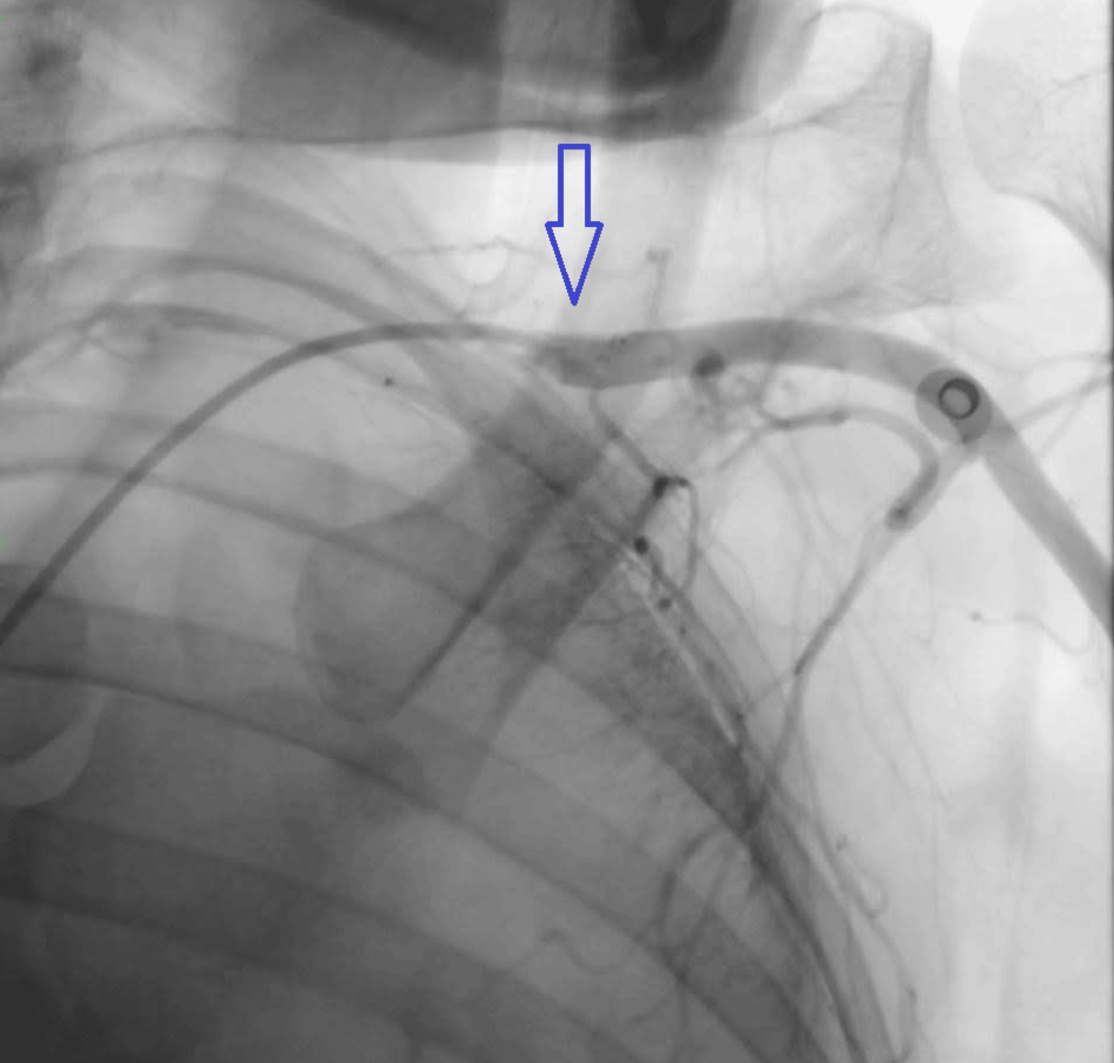
Arterial embolization of internal mammary artery

The patient, having defaulted for 15 days, returned with distressing symptoms of dyspnea and chest pain. A repeat assessment, including a second β-hCG test, revealed a signiﬁcantly elevated β-hCG level of 3,79,100 IU/ml, indicating a concerning progression. The patient was given etoposide and cisplatin chemotherapy. Within 24 hours post-chemotherapy administration, the patient experienced a notable decline in hemoglobin levels accompanied by the onset of tachycardia. As the day progressed, a chest X-ray revealed opacity in the left hemithorax. As these tumors are highly vascular, the patient underwent embolization of the left internal mammary artery (IMA) and branches of the left subclavian vein (Figures 5, 6).

**Figure 7:**
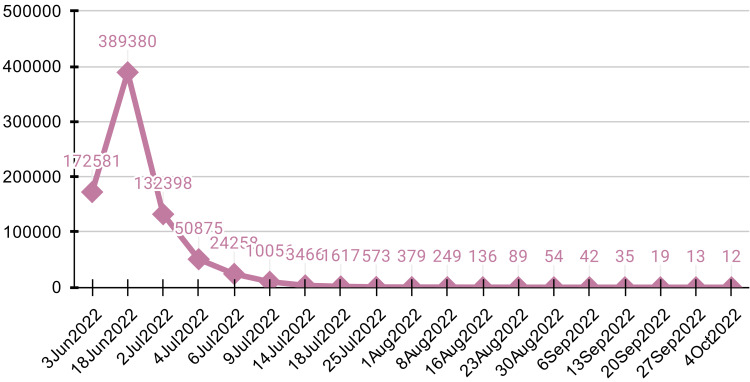
Graph showing a gradual fall of β-hCG with treatment Beta-HCG in mIU/mL versus date β-hCG = beta-human chorionic gonadotropin

Over three days, 500 ml of hemorrhagic ﬂuid was tapped from the intercostal drainage tube (ICD), underscoring ongoing complications. The patient received the second dose of chemotherapy. Following a gradual decline in β-hCG levels (Figure 7), the ICD was successfully removed on day 10, leading to the patient's discharge on day 11. The third dose of chemotherapy was administered on days 14 and 15. 

Subsequently, the patient received cycles of chemotherapy EMA-CO regimen (etoposide, methotrexate, actinomycin D, cyclophosphamide, vincristine) in a 14-day cycle (Table 1). The patient showed a complete response. Currently, after nine months of treatment, there is no sign of disease or recurrence. 

**Table 1:** Chemotherapy regimen

Drugs	Doses	Days
Dactinomycin (Actinomycin D)	0.5 mg	1 and 2
Etoposide	100 mg/m^2^	1 and 2
Methotrexate	300 mg/m^2^	1
Vincristine	1 mg/m^2^	8
Cyclophosphamide	600 mg/m^2^	8

## Discussion

Choriocarcinoma is a rare and highly aggressive germ cell tumor, characterized by the presence of syncytiotrophoblast cells and the secretion of β-hCG hormone. Choriocarcinoma poses significant diagnostic and therapeutic challenges due to its rapid progression and propensity for early metastasis [3]. PPC is an exceptionally rare malignancy believed to originate from primordial germ cells that migrate to the lung during embryogenesis.

The unique histopathological features of choriocarcinoma, including the presence of both cytotrophoblast and syncytiotrophoblast elements, contribute to its distinct clinical behavior. Unlike its more common gonadal counterparts, PPC is an entity that demands heightened clinical awareness, considering its scarcity and potential diagnostic pitfalls [4]. These tumors stand out due to their highly malignant nature and notorious reputation for an exceedingly poor prognosis. They often metastasize to distant organs such as bone, liver, brain, spleen, and contralateral lung [4]. With a five-year survival rate that consistently hovers below 5%, the clinical management of patients afflicted by these tumors presents formidable challenges to healthcare professionals [3,5].

The underlying mechanisms leading to the development of primary choriocarcinoma in the lung remain unclear. A few hypotheses that could lead to the development of choriocarcinoma from the lung are mentioned here. PPC may represent metastasis from primary gonadal choriocarcinoma that regressed spontaneously [6,7], or primordial germ cells that migrate to the lung during embryogenesis [7-9]. Lung cancer that develops originally as non-trophoblastic neoplasm and later de-differentiates [9]. The origin of PPC is from trophoblastic embolism related to gestational events after a long period of latency [10,11]. The clinical presentation of PPC shares similarities with other lung cancers. Patients commonly exhibit respiratory symptoms, including a persistent cough, chest pain, and dyspnea. However, the distinctive nature of this condition may also lead to symptoms attributable to the compression or invasion of the tumor into the surrounding structures. Hemoptysis is a notable manifestation, highlighting the high vascularity of the tumor and the associated life-threatening risk of heavy bleeding [11].

Additionally, patients may experience symptoms related to the tumor's impact on nearby structures, such as dysphagia and manifestations of superior vena cava syndrome. This can result in symptoms like swelling of the face, neck, and upper extremities, along with headache and altered consciousness. The diagnostic criteria involve excluding previous gynecologic malignancy, conﬁrming a solitary or predominant lung lesion, and assessing elevated serum β-hCG levels that normalize post-surgery or chemotherapy [12]. Pathologic confirmation through tissue biopsy or surgical resection is essential for a deﬁnitive diagnosis. Distinguishing PPC from giant cell carcinoma, another lung malignancy producing β-hCG, required careful consideration. Unlike in giant cell carcinoma, the absence of thyroid transcription factor 1 (TTF-1) immunoreactivity in PPC facilitated accurate differentiation [9,12,13]. Poor prognostic factors include male gender, smoking, age more than 40 years, tumor size ≥5 cm, and the presence of metastasis [2,12].

We present a unique case where metastasis occurred from a spontaneously regressed primary gonadal choriocarcinoma. Despite lacking a history of gynecologic malignancy, our patient exhibited classical respiratory symptoms associated with PPC, including cough, chest pain, and dyspnea. Notably, the tumor's high vascularity led to life-threatening complications such as hemothorax. Diagnosing PPC posed challenges due to its clinical similarities with other lung cancers. Elevated serum β-hCG titers normalized post-treatment and served as a crucial diagnostic marker. With no official treatment guidelines for PPC, our patient received a combination of chemotherapy, demonstrating favorable outcomes. Chemotherapeutic regimens such as cisplatin-etoposide and EMA-CO were employed, and selective arterial embolization proved crucial for managing life-threatening bleeding. This case highlights the diagnostic and therapeutic challenges associated with PPC, an exceedingly rare pulmonary malignancy with distinct embryogenic origins and potential for spontaneous regression.

## Conclusions

PPC is an exceptionally rare condition characterized by diverse clinical features and outcomes. A multidisciplinary approach integrating surgery, chemotherapy, and interventional procedures is essential for its effective management. Ongoing research is needed to enhance our understanding of PPC, refine diagnostic criteria, and establish optimal treatment strategies for improved patient outcomes.
